# Estimates of Treatable Deaths Within the First 20 Years of Life from Scaling Up Surgical Care at First-Level Hospitals in Low- and Middle-Income Countries

**DOI:** 10.1007/s00268-022-06622-w

**Published:** 2022-06-30

**Authors:** Alicia G. Sykes, Justina Seyi-Olajide, Emmanuel A. Ameh, Doruk Ozgediz, Alizeh Abbas, Simone Abib, Adesoji Ademuyiwa, Abdelbasit Ali, Tasmiah Tahera Aziz, Tanvir Kabir Chowdhury, Hafeez Abdelhafeez, Romeo C. Ignacio, Benjamin Keller, Greg Klazura, Karen Kling, Benjamin Martin, Godfrey Sama Philipo, Hariharan Thangarajah, Ava Yap, John G. Meara, Donald A. P. Bundy, Dean T. Jamison, Charles N. Mock, Stephen W. Bickler, On behalf of the Global Initiative for Children’s Surgery

**Affiliations:** 1 Naval Medical Center San Diego, San Diego, CA USA; 2 Pediatric Surgery Unit, Lagos University Teaching Hospital, Lagos, Nigeria; 3 Division of Pediatric Surgery, Department of Surgery, National Hospital, Abuja, Nigeria; 4 Division of Pediatric Surgery, Department of Surgery, University of California San Francisco, San Francisco, CA USA; 5 The Aga Khan University, Karachi, Pakistan; 6 Paulista School of Medicine, Federal University of São Paulo, São Paulo, Brazil; 7 Department of Surgery, College of Medicine, University of Lagos, Lagos, Nigeria; 8 University of Khartoum, Khartoum, Sudan; 9 Chittagong Research Institute for Children Surgery, Chittagong, Bangladesh; 10 St. Jude Children’s Research Hospital, Memphis, TN USA; 11 Division of Pediatric Surgery, Department of Surgery, University of California San Diego School of Medicine, 9500 Gilman Drive #0739, La Jolla, San Diego, CA 92093-0739 USA; 12 Loyola University Medical Center, Chicago, IL USA; 13 Department of Paediatric Surgery and Urology, Bristol Children’s Hospital, Bristol, UK; 14 Muhimbili University of Health and Allied Sciences, Dar es Salaam, Tanzania; 15 Program in Global Surgery and Social Change, Harvard Medical School, Boston, MA USA; 16 Global Research Consortium for School Health and Nutrition, London School of Hygiene and Tropical Medicine, London, UK; 17 Institute for Global Health Sciences, University of California, San Francisco, San Francisco, CA USA; 18 University of Washington, Seattle, WA USA

## Abstract

**Background:**

Surgical care is an important, yet often neglected component of child health in low- and middle-income countries (LMICs). This study examines the potential impact of scaling up surgical care at first-level hospitals in LMICs within the first 20 years of life.

**Methods:**

Epidemiological data from the global burden of disease 2019 Study and a counterfactual method developed for the disease control priorities; 3rd Edition were used to estimate the number of treatable deaths in the under 20 year age group if surgical care could be scaled up at first-level hospitals. Our model included three digestive diseases, four maternal and neonatal conditions, and seven common traumatic injuries.

**Results:**

An estimated 314,609 (95% UI, 239,619–402,005) deaths per year in the under 20 year age group could be averted if surgical care were scaled up at first-level hospitals in LMICs. Most of the treatable deaths are in the under-5 year age group (80.9%) and relates to improved obstetrical care and its effect on reducing neonatal encephalopathy due to birth asphyxia and trauma. Injuries are the leading cause of treatable deaths after age 5 years. Sixty-one percent of the treatable deaths occur in lower middle-income countries. Overall, scaling up surgical care at first-level hospitals could avert 5·1% of the total deaths in children and adolescents under 20 years of age in LMICs per year.

**Conclusions:**

Improving the capacity of surgical services at first-level hospitals in LMICs has the potential to avert many deaths within the first 20 years of life.

**Supplementary Information:**

The online version contains supplementary material available at 10.1007/s00268-022-06622-w.

## Introduction

Over the past twenty years, significant improvement has been made in child health worldwide; however, most efforts have focused on children under the age of five years [1–4]. As under-five mortality has fallen, there has been a growing recognition that the healthcare needs of older children must also be addressed. An evolving theme has been to focus on the first 8000 days^1^ of life [5, 6]. This time represents a critical period of growth and development as children transition to adulthood. Recent evidence suggests that the morality of children 5–19 years of age is substantially higher than previously thought, and increasing efforts are needed to recognize and appropriately prioritize health interventions to decrease morbidity and mortality in this age group [5, 7].

Surgical care is a critical, yet often overlooked, component of child health [8]. During the first 8000 days of life, surgical care can reduce the short- and long-term suffering associated with congenital anomalies, traumatic injuries, and childhood malignancies amenable to surgery. Early surgical treatment of debilitating conditions can allow children to obtain an education, which directly influences a child’s overall development, and can improve a child’s chances of survival. In addition, surgical care can play a role in achieving health-related sustainable development goals and targets, in particular: (1) ending preventable deaths in newborn babies and children younger than 5 years; (2) reducing death and disability due to road traffic injuries and noncommunicable diseases; (3) ensuring universal health coverage; and (4) increasing the health workforce [9].

Despite the established benefits of surgery, there continue to be major gaps in surgical services worldwide. Globally, an estimated 1.7 billion children and adolescents do not have access to basic, lifesaving, and safe surgical care [10]. This lack of access to surgical care disproportionately affects low- and middle-income countries (LMICs), where children make up 50–60% of the population. With less than 8% of children living in LMICs having access to safe, affordable, and timely surgical care, an important opportunity exists to improve child health by scaling up surgical care in the poorest regions of the world [10].

First-level hospitals represent a critical platform for the delivery of surgical care in LMICs [11]. A large percentage of the world’s population receives emergency care at first-level facilities due to their proximity to rural and under-served populations. Additionally, many surgical conditions—particularly obstetric emergencies, intra-abdominal catastrophes, and life-threatening injuries—require immediate and appropriate lifesaving surgical care [12]. Surgical care delivered at first-level hospitals in LMICs can be one of the most cost-effective components of a public health system [13]. An estimated 1.4 million deaths per year could be averted if basic surgical care was universally available at first-level hospitals in LMICs [11]. In this study, we estimate the number of treatable deaths [14]^2^ in the under-20 age group if surgical care was universally accessible at first-level hospitals in LMICs.

## Methods

Data from the 2019 Global Burden of Diseases (GBD) Study [15] was used to estimate the number of deaths that could be averted in the under-20 year age group by scaling up surgical care provided at first-level hospitals. Our approach was similar to that used to estimate the number of preventable surgical deaths in Chapter 2 of Disease Control Priorities, 3rd Edition (DCP3), Volume 1: Essential Surgery [12]. This methodology assumes a basic surgical package^3^ with various therapeutic interventions that can be provided at first-level hospitals [16–18]. Our model included the following, with 2019 GBD cause codes shown in parenthesis:

- *Three digestive diseases*^4^ appendicitis (B.4.3); paralytic ileus and intestinal obstruction (B.4.4); inguinal, femoral, and abdominal hernia (B.4.5).
- *Four maternal and neonatal conditions (A.6)* maternal hemorrhage (A.6.1.1), maternal obstructed labor and uterine rupture (A.1.1.4), maternal abortion and miscarriage (A.1.1.5), and neonatal encephalopathy due to birth asphyxia and trauma (A.6.2.2).
- *Eight injuries that could be treated with basic interventions* adverse effects of medical treatment (C.2.6)^5^; exposure to mechanical forces (C.2.5); falls (C.2.1); fire, heat, and hot substances (C.2.3); interpersonal violence (C.3.2); non-venomous animal contact (C.2.7.2); road injuries (C1.1); and other transport injuries (C.1.2).

This approach recognizes that some conditions, such as maternal hemorrhage and neonatal encephalopathy, are not entirely amenable to surgical care and hence require adjustments to limit the effect of surgery [12, 19]. Adjustments for the effect of surgical care were based on information provided in Annex 2E of Chapter 2 of the Essential Surgery Volume of DCP3 [12] and are included in Additional file 1 of the supplemental information.

The overall concept of our approach was to split the reported deaths from surgical conditions into surgically treatable and non-treatable deaths. Treatable deaths were calculated as follows:

1 $${{TREATABLE\,DEATHS}} = {{DEATHS}}^{{current}} - {{DEATHS}}^{counterfactual}$$

where *DEATHS*^*current*^ denotes the deaths reported in GBD 2019, and *DEATHS*^*counterfactual*^ represents the estimated number of deaths if delivery of surgical care had existed in a “counterfactual” state, which is described as the state in which the entire population has access to appropriate and safe surgical care deliverable at first-level hospital.

To make these calculations, we downloaded age and income-specific death rates, uncertainty intervals, and population data from the Institute of Health Metrics and Evaluation using the GBD Results tool [20]. The treatable death rates for the World Bank LMIC income groups (low-income, lower-middle-income and upper middle income) were calculated by subtracting the cause-specific high-income death rates from the cause-specific rates in the low- and middle-income income groupings using the following formula:

2 $${{ADR}}_{age\,group}^{{income\,group}} = {{EDR}}_{age\,group}^{income\,group} - {{CDR}}_{age\,group}^{income\,group}$$

where ${{ADR }}_{age\,group}^{income\,group}$ is the cause-specific treatable death rate for each age and income group, ${{EDR}}_{age\,group}^{income\,group}$ the existing cause-specific death rates reported in GBD 2019, and ${{CDR}}_{age\,group}^{income\,group}$ the cause-specific death rates for the counterfactual state. We assumed that the lowest fatality rate to be in the high-income group and therefore representative of ${{CDR}}_{age\,group}^{{income\,group}}$. The number of treatable deaths for each age and income group was determined by multiplying the cause-specific treatable death rates by the population in each category (Additional file 2). Finally, we corrected for the effect of surgical care and variability in access by multiplying the number of treatable deaths times the correction factors listed in Additional file 1.

## Results

**Fig. 1:**
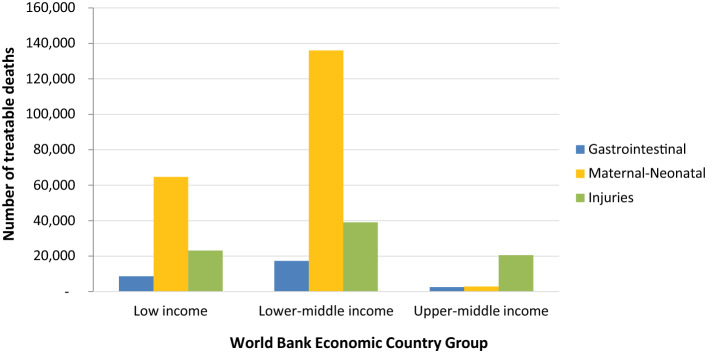
Distribution of treatable deaths from scaling up surgical care at first-level hospitals in LMICs by World Bank economic groups

Scaling up surgical care at first-level hospitals in LMICs could avert an estimated 314,609 (95% UI, 239,619–402,005) child and adolescent deaths per year. Details of these treatable deaths by World Bank income groupings are shown in Table 1, Fig. 1, and Additional file 3. Most of the treatable deaths occur in lower middle-income (192,371 deaths; 61.1%) and low-income countries (96,353 deaths; 30.6%). The largest number of treatable deaths in the under 20 years age group are from improving surgical care for maternal conditions (64.7%), followed by injuries (26.3%) and digestive diseases (9.0%).

**Table 1:** Estimated number of treatable deaths within the first 20 years of life from scaling up surgical care at first-level hospital in LMICs by Global Burden of Disease (GBD) cause and World Bank economic groups

GBD cause		Low-income	Lower middle income	Upper middle income	Total LMIC	% of Category	% of Total
GI	Appendicitis	1326 (613–2,054)	3,040 (2,192–4,040)	385 (311–489)	4751 (3116–6584)	16.7	1.5
	Inguinal, femoral, and abdominal hernia	1079 (572–1612)	1423 (2192–4040)	150 (109–222)	2652 (1667–3766)	9.3	0.8
	Paralytic ileus and intestinal obstruction	6152 (4246–9070)	12,851 (7941–19,312)	2008 (1544–2564)	21,012 (13,742–30,946)	73.9	6.7
	GI Total	8557 (5,431–12,736)	17,315 (11,120–25,285)	2543 (1,974–3,275)	28415 (18,525–41,296)	100.0	9.0
Maternal- Neonatal	Maternal abortion and miscarriage	1332 (1,011–1,700)	731 (536–1,010)	44 (35–53)	2107 (1,583–2,763)	1.0	0.7
	Maternal hemorrhage	339 (264–420)	1082 (830–1,393)	36 (32–43)	1457 (1,125–1,856)	0.7	0.5
	Maternal obstructed labor and uterine rupture	282 (205–382)	537 (378–741)	28 (22–37)	848 (605–1160)	0.4	0.3
	Neonatal encephalopathy due to birth asphyxia and trauma	62754 (48,926–78,779)	133,588 (111,933–157,461)	2747 (2,353–3,204)	199,088 (16,3211–239,443)	97.8	63.3
	Maternal-neonatal total	64,707 (50,406–81,280)	135,938 (113,676–160,605)	2856 (2442–3337)	203,500 (166,524–245,222)	100.0	64.7
Injury	Adverse effects of medical treatment	4335 (2,789–6494)	5047 (3,594–6813)	548 (431–723)	9,930 (6,814–14,030)	12.0	3.2
	Exposure to mechanical forces	1688 (807–2861)	3313 (1,533–5076)	1882 (1,197–2351)	6884 (3,538–10,289)	8.3	2.2
	Falls	1543 (942–2328)	6409 (4243–8734)	3582 (2518–4265)	11,534 (7704–15,327)	13.9	3.7
	Fire, heat, and hot substances	3366 (2156–5098)	6498 (3542–9912)	831 (516–1211)	10,695 (6215–16,221)	12.9	3.4
	Interpersonal violence	1750 (994–2566)	1398 (284–2601)	4846 (4274–5471)	7993 (5552–10,638)	9.7	2.5
	Non-venomous animal contact	724 (490–1091)	528 (376–743)	86 (68–110)	1338 (934–1944)	1.6	0.4
	Road injuries	9278 (6110–14,051)	14,720 (9696–20,995)	8684 (7183–10,442)	32,682 (22,990–45,488)	39.5	10.4
	Other transport injuries	405 (160–700)	1205 (667–1,685)	28 (− 4.6–64.7)	1638 (823–2,450)	2.0	0.5
	Injury total	23,089 (14,451–35,189)	39,118 (23,935–56,561)	20,487 (16,183–24,637)	82,694 (54,569–116,387)	100.0	26.3
Total number of avertable deaths		96,353 (70,288–129,205)	192,371 (148,732–242,451)	25,885 (20,599–31,249)	314,609 (239,619–402,905)	100.0	100.0
% of total by world bank groupings		30.6	61.1	8.2	100.0	–	–

Lower and upper 95% Uncertainty Intervals are shown in paratheses. See Additional file 1 for information on number of treatable deaths by age groups

Improving the surgical care to treat four common maternal causes at first-level hospitals in LMICs could avert 203,500 (95% UI, 166,524–245,222) child and adolescent deaths per year. The most common causes of these treatable deaths are neonatal encephalopathy due to birth asphyxia and trauma (199,088 deaths; 97.8%), followed by maternal abortion and miscarriage (2107 deaths; 1.0%), and maternal hemorrhage (1457 deaths; 0.7%). Most of these treatable deaths occur in lower middle- (66.8%) and low-income countries (31.8%).

Improving the surgical care to treat eight common injuries in LMICs could avert 82,694 (95% UI, 54,569–116,387) child and adolescent deaths per year. The most common causes of these treatable deaths are road injuries (32, 682; 39.5%), followed by falls (11,534 deaths; 13.9%), and fire, heat, and hot substances (10,695 deaths; 12.9%). Most of these treatable deaths occur in lower middle- (47.3%) and low-income countries (27.9%).

Improving surgical care to treat three common gastrointestinal causes in LMICs could avert 28,415 (95% UI, 18,525–41,296) child and adolescent deaths per year. The most common causes of these treatable deaths are paralytic ileus and intestinal obstruction (21,012 deaths; 73.9%), followed by appendicitis (4751 deaths; 16.7%). Like the maternal and neonatal conditions, most of these treatable deaths occur in lower middle- (60.9%) and low-income countries (30.1%).

**Fig. 2:**
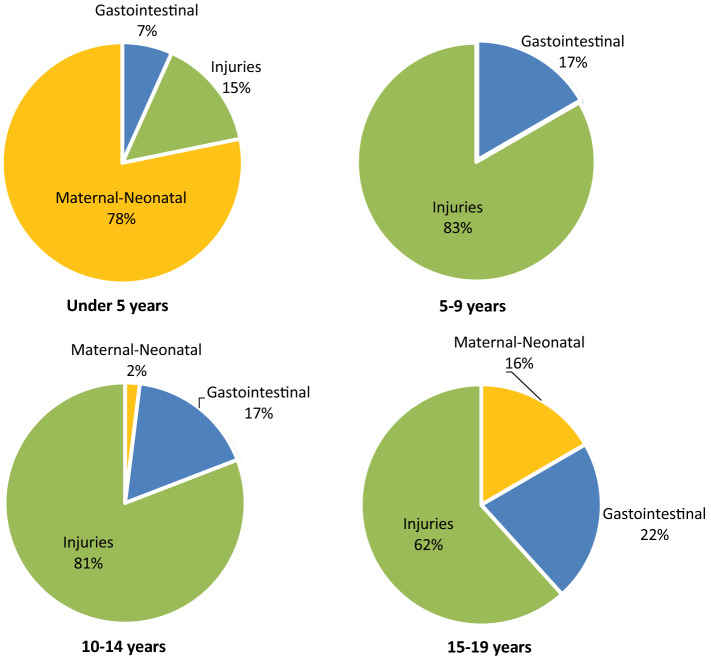
Distribution of treatable deaths from scaling up surgical care at first-level hospitals in LMICs by age groups

The causes of the treatable deaths from scaling up surgical care at first-level hospitals in LMICs vary by age (Fig. 2). Within the under-five age group, the largest number of treatable deaths are from scaling up surgical care to address maternal and neonatal causes (78%). Treatable deaths from maternal causes increase in the 10–14- and 15–19-years age groups (2% and 16%, respectively). Injuries and gastrointestinal causes are represented across the span of childhood, with injuries accounting for the largest fraction of the treatable deaths over the age of 5 years. Within the 5–9 and 10–14 age groups injuries accounted for 83% and 81% of the treatable deaths, respectively.

Overall, scaling up surgical care at first-level hospitals could avert 5.1% of the total deaths in children and adolescents under 20 years of age in LMICs (Table 2). The majority (80.9%) of these treatable deaths would be in the under-five year age group, mainly due to improvements in obstetrical care with its effect on reducing neonatal encephalopathy due to birth asphyxia and trauma.

**Table 2:** Fraction of total deaths that would be averted by scaling up surgical care at first-level hospitals in LMICs

	World bank low income			World bank lower middle income			World bank upper middle income			LMIC total		
Age group	Treatable deaths	Total deaths	%	Treatable deaths	Total deaths	%	Treatable deaths	Total deaths	%	Treatable deaths	Total deaths	%
Under 5	81,878	1,651,643	5.0	162,394	2,911,796	5.6	10,312	419,948	2.5	254,583	4,983,387	5.1
5–9 years	5093	100,908	5.0	10,262	223,070	4.6	4,422	47,288	9.4	19,776	371,266	5.3
10–14 years	3735	68,677	5.4	8513	178,221	4.8	3298	44,495	7.4	15,546	291,393	5.3
15–19 years	5647	100,901	5.6	11,202	274,138	4.1	7854	99,917	7.9	24,703	474,956	5.2
	96,353	1,922,129	5.0	192,371	3,587,225	5.4	25,886	611,648	4.2	314,608	6,121,003	5.1

## Discussion

In this study, we used published GBD 2019 data and a counterfactual method developed for DCP3 to estimate the number of treatable child and adolescent deaths from scaling surgical care at first-level hospitals in LMICs. Our analysis showed that scaling up surgical care at first-level hospitals in LMICs could avert almost 315,000 deaths of children and adolescents per year. This number of deaths represents approximately 22% of the 1.4 million deaths per year that were estimated by DCP3 should surgical care be scaled up at first-level hospitals to treat all age groups.

Perhaps, the most important finding of our study was that improving surgical care for pregnant females at first-level hospital in LMICs could have a profound effect on the under 20 mortality rate. Almost two-thirds of the treatable surgical deaths in the under 20 years age group were attributable to improvements in surgical care directed at pregnant females. This relationship exists because stillbirths and intrapartum-related neonatal deaths are often associated with difficult and obstructed labor [21]. Thus, provision of assisted vaginal delivery and Caesarian delivery are vital to the reduction of perinatal morbidity and mortality. The importance here is that a substantial reduction in the neonatal death rate is possible via surgical interventions in the mother, rather than direct intervention in the neonate.

To further explore the relationship between improved surgical care for pregnant females at first-level hospital in LMICs and the under 20 years mortality rate, we calculated the fraction of the total maternal and neonatal deaths (GBD cause category A.6) that would be averted in the under 20 years age group. The 2019 GBD study reported 19,865 LMICs deaths in the under 20 years age group from maternal causes (Additional file 4). Twenty-two percent of these (4412 deaths) would be averted by scaling up surgical care at first-level hospitals in LMICs. Considering only the neonatal encephalopathy due to birth asphyxia and trauma cause category, 199,088 of 562,556 (35.3%) deaths could be averted. Thus, within specific GBD cause categories, basic surgical care delivered at first-level hospitals could be an important strategy for reducing the under-five mortality rate, and especially neonatal deaths.

Our analysis also suggests there is an important opportunity to reduce the under 20 death rates in LMICs by improving the surgical care of injuries. Treatable deaths related to injuries occurred across the span of the first 8000 days of life and account for the largest fraction of treatable deaths in the 5–19 years age group. Almost, one-third of these treatable injury deaths are due to road injuries. Road traffic injuries have long been known to be a major cause of death and disability worldwide, with the majority of deaths in LMICs occurring in pedestrians, cyclists, and children [22]. As UN SDG 3.6 is aimed at reducing the number of global deaths and injuries from road traffic accidents, improving surgical care within LMICs can play an important role in achieving this goal.

Treatable deaths from scaling up surgical care to treat gastrointestinal causes were considerably less than the number of treatable deaths in the maternal-neonatal and injury categories. This may reflect that fewer GBD causes were examined in the gastrointestinal category. However, it is more likely that gastrointestinal conditions are less common in children relative to deaths associated with maternal and neonatal conditions and injuries [23, 24]. Nevertheless, surgical treatment of common gastrointestinal conditions is an attractive intervention for the first-level hospital as they are lifesaving, relatively easy to perform, restore health quickly and are cost-effective.

An unexpected finding of our study was that the largest number of treatable deaths from scaling up surgical care at first-level hospitals would occur in lower-middle-income, rather than in low-income countries. This occurs because the under 20 years population in lower middle-income countries is more than three times larger than the under 20 years population in the low-income countries (Additional file 2). This finding disguises the fact that the situation is worse in low-income countries (i.e., people living in LICs are more likely to die from a treatable surgical death than anywhere else). Perhaps the good news is that lower-middle-income countries have the extra fiscal space, compared to low-income countries, to scale up actions in response to recognized needs. As an example, national school meals programs increased coverage over the last 10 years by 86% in lower middle-income countries and only 36%, despite best efforts, in low-income countries [25]. This implies that the largest treatable burden is in countries that have the greater potential to respond. It doesn’t solve the problems for the worst affected countries, but it would save a substantial number of lives.

As our analysis included only 13 of the 369 GBD causes, it is important that our estimate not be interpreted as the total number of child and adolescent deaths that could be averted if surgical care would be scaled up across all levels of the health care system. Important GBD cause categories not included in our analysis were congenital anomalies and cancer. Birth defects are now the fifth most common cause of death in children younger than 5 years with many congenital anomalies correctable with surgery [26, 27]. In 2018, there were over 200,000 cases of childhood cancers accounting for almost 75,000 deaths [28]. Beyond preventing death, surgical care also has a role in the diagnosis and treatment of a wide variety of other healthcare problems [29, 30].

Our study has several limitations. First, it shares the limitations of the overall GBD approach [15, 31], including it being a descriptive study; limitations in data availability (e.g., reporting lags, disruptions in settings with conflict, natural disasters, or domestic governance crises); variable data granularity with respect to age and cause detail; varying quality and completeness of mortality reporting systems; and the core GBD assumption of each death having only a single underlying cause. Second, our analysis required assumptions to be made about the effect of surgery and access to health systems. As emphasized in Chapter 2 of the Essential Surgery Chapter DCP3 [12], estimating the effect of surgical care is challenging as the effectiveness of an operation varies by the type of operation; resources available to conduct the operation; operative skills of the surgeon; capability, and resources of anesthesia personnel; and patient factors, such as nutritional status, age and other comorbidities. For consistency, we used the adjustments for the effect of surgery and access reported in Annex 2E of the Essential Surgery Chapter of DCP3 [12]. These adjustments are based on a combination of factors that best represent the variable effect surgical care can have on health conditions. As an example, the adjustment for the effect of surgery on neonatal encephalopathy due to birth asphyxia and trauma was based on the 40% risk reduction that was used in the World Health Organization Choosing Interventions that are Cost Effective (WHO CHOICE) project [32–34]. Because applying this assumption uniformly to all regions leads to an overestimation of treatable deaths, the effect was also scaled for access to healthcare. Third, our model assumes that healthcare access is equal across all age groups, which may be problematic given the variability in importance that children occupy in society in different regions of the world. Fourth, while we have described our estimates as the number of deaths that could be prevented if surgical care would be scaled up at first-level hospitals, more accurately we have estimated the number of treatable deaths from scaling up a basic surgical package across all levels of the healthcare system. Nevertheless, our estimates still likely approximate the impact of scaling up surgical care at first-level hospitals because most gaps in surgical care exist in geographic areas served by first-level hospitals and most higher-level facilities in LMICs can provide the surgical care our model. Finally, although our framework for estimating treatable surgical deaths is conceptually simple, and a powerful tool for estimating the impact of scaling up surgical care in LMICs, it is also limited by its inherently retrospective nature, and its inability to parse competing risks or factors that might influence geographical variability in surgical care.

In conclusion, our findings suggest an important opportunity exists to improve the health of children and adolescents living in LMICs by scaling up basic surgical care at first-level hospitals. As much of care is directed at women of child-bearing age, these efforts would have a dual benefit—directly improving the health of women and indirectly reducing the deaths of infants associated with obstructed labor. Future research should focus on confirming the intrinsic link between improving obstetrical care at first-level hospitals and infant survival and defining the impact of scaling up surgical care that is beyond the scope of the first-level hospital.

## Supplementary Information

Below is the link to the electronic supplementary material.

Supplementary file1 (DOCX 9 KB)

Supplementary file2 (DOCX 8 KB)

Supplementary file3 (DOCX 19 KB)

Supplementary file4 (DOCX 8 KB)

## Data Availability

The dataset analyzed and the excel spread sheets for the analysis are available upon reasonable request from the corresponding author: sbickler@health.ucsd.edu.

## Footnotes

1 The first 8000 days of life is equivalent to 21.9 years. Because data is collected in 5 year increments, our focus was on the under < 20 year age group.

2 Treatable (or amenable) mortality refers to causes of death that can be mainly avoided through timely and effective health care interventions [14].

3 Almost 50 surgical procedures are required to treat the 2019 GBD causes included in our model. See Annex 2D of Chapter 2 of the Essential Surgery Volume of DCP3 for a list of these surgical procedures [12].

4 Gallbladder and bile duct disease, which was included in the DCP3 estimates, was not included in our analysis due the low incidence of these conditions in the under 20 years age group.

5 Death or short-term or long-term disability sustained as the result of undergoing a procedure, treatment, or other exposure to the healthcare system [18].
