# Supplementary material for: Estimates of Treatable Deaths Within the First 20 Years of Life from Scaling Up Surgical Care at First-Level Hospitals in Low- and Middle-Income Countries
Source: World J Surg. 2022 Jun 30;46(9):2114–22. doi: 10.1007/s00268-022-06622-w (PMC9334432; doi:10.1007/s00268-022-06622-w)
Supplement: Supplementary file 1 — Supplementary file1 (DOCX 9 KB) [file 268_2022_6622_MOESM1_ESM.docx]

Additional file 1_Adjustments to account for deaths not treatable by surgical care

**Major cause Cause name Low-income Lower-middle-income Upper-middle income**

GI Appendicitis 1.0 1.0 1.0

Inguinal, femoral, and abdominal hernia 1.0 1.0 1.0

Paralytic ileus and intestinal obstruction 1.0 1.0 1.0

Injury Adverse effects of medical treatment 0.845 0.885 0.915

Exposure to mechanical forces 0.31 0.435 0.45

Falls 0.47 0.37 0.71

Fire, heat, and hot substances Interpersonal violence

| 0.58 | 0.665 | 0.6 |
| --- | --- | --- |
| 0.27 | 0.33 | 0.32 |
| 0.285 | 0.265 | 0.575 |

Non-venomous animal contact

| 1.0 | 1.0 | 1.0 |
| --- | --- | --- |
| 0.36 | 0.36 | 0.36 |

Maternal- Neonatal

| Other transport injuries | 0.275 | 0.395 | 0.285 |
| --- | --- | --- | --- |
| Road injuries | 0.40 | 0.545 | 0.49 |

Maternal abortion and miscarriage Maternal hemorrhage

| Maternal obstructed labor and uterine rupture  Neonatal encephalopathy due to birth asphyxia and | 1.0 | 1.0 | 1.0 |
| --- | --- | --- | --- |
| trauma | 0.39 | 0.4 | 0.1 |

Adjustments for the effect of surgical care were based on information provided in Annex 2D of Chapter 2 of the Essential Surgery Volume of DCP3 (Reference 10). When adjustments for the effect of surgery and access varied between the sexes, values were averaged.
