# Supplementary material for: Estimates of Treatable Deaths Within the First 20 Years of Life from Scaling Up Surgical Care at First-Level Hospitals in Low- and Middle-Income Countries
Source: World J Surg. 2022 Jun 30;46(9):2114–22. doi: 10.1007/s00268-022-06622-w (PMC9334432; doi:10.1007/s00268-022-06622-w)
Supplement: Supplementary file 2 — Supplementary file2 (DOCX 8 KB) [file 268_2022_6622_MOESM2_ESM.docx]

Additional file 2_Population data for the under 20 years age group

| **location**  **_id** | **location_name** | **age_group_n** | **val** | **lower** | **upper** | **totals for different age groups** |
| --- | --- | --- | --- | --- | --- | --- |
| **44575** | World Bank High Income | Under 5 | 61,871,619.4 | 59,235,516.6 | 64,684,269.8 |  |
| **44575** | World Bank High Income | 5 to 9 | 65,084,448.7 | 62,375,357.1 | 67,971,447.9 |  |
| **44575** | World Bank High Income | 10 to 14 | 67,145,682.7 | 64,202,727.3 | 70,222,996.7 |  |
| **44575** | World Bank High Income | 15 to 19 | 67,976,428.1 | 64,971,362.7 | 71,050,991.7 | 262,078,178.9 |
| **44576** | World Bank Upper Middle Income | Under 5 | 174,229,203.0 | 163,446,866.9 | 184,830,524.5 |  |
| **44576** | World Bank Upper Middle Income | 5 to 9 | 166,905,397.7 | 157,155,496.9 | 176,531,894.5 |  |
| **44576** | World Bank Upper Middle Income | 10 to 14 | 163,093,847.8 | 153,646,387.5 | 172,446,431.3 |  |
| **44576** | World Bank Upper Middle Income | 15 to 19 | 166,037,494.7 | 156,009,358.5 | 175,813,615.5 | 670,265,943.2 |
| **44577** | World Bank Lower Middle Income | Under 5 | 315,800,258.5 | 302,036,616.8 | 330,459,716.8 |  |
| **44577** | World Bank Lower Middle Income | 5 to 9 | 322,590,147.7 | 307,795,351.3 | 338,011,176.3 |  |
| **44577** | World Bank Lower Middle Income | 10 to 14 | 322,447,902.2 | 307,118,983.1 | 338,763,623.1 |  |
| **44577** | World Bank Lower Middle Income | 15 to 19 | 307,497,261.9 | 292,004,659.9 | 323,763,171.3 | 1,268,335,570.3 |
| **44578** | World Bank Low Income | Under 5 | 110,514,626.7 | 104,768,855.7 | 116,067,814.7 |  |
| **44578** | World Bank Low Income | 5 to 9 | 99,710,899.9 | 94,436,477.8 | 104,736,622.6 |  |
| **44578** | World Bank Low Income | 10 to 14 | 89,104,636.0 | 84,434,005.7 | 93,529,279.7 |  |
| **44578** | World Bank Low Income | 15 to 19 | 77,650,058.7 | 73,653,933.2 | 81,476,883.9 | 376,980,221.4 |
|  |  |  | **2,577,659,913.8** | **2,447,291,956.8** | **2,710,360,460.3** | **2,577,659,913.8** |
