# Supplementary material for: Estimates of Treatable Deaths Within the First 20 Years of Life from Scaling Up Surgical Care at First-Level Hospitals in Low- and Middle-Income Countries
Source: World J Surg. 2022 Jun 30;46(9):2114–22. doi: 10.1007/s00268-022-06622-w (PMC9334432; doi:10.1007/s00268-022-06622-w)
Supplement: Supplementary file 3 — Supplementary file3 (DOCX 19 KB) [file 268_2022_6622_MOESM3_ESM.docx]

**Additional file 3.** Estimated number of avertable deaths from scaling up surgical care at first-level hospital in LMICs by GBD cause, age, and World Bank economic groups. Lower and upper refer to 95% Uncertainty Intervals.

| **GBD Cause** | | **Age (years)** | **Low-income** | | | **Lower-middle income** | | | **Upper-middle-income** | | | **Total LIMC** | | | **% of Cat** | **% of Total** |
| --- | --- | --- | --- | --- | --- | --- | --- | --- | --- | --- | --- | --- | --- | --- | --- | --- |
|  |  |  | **Value** | **Lower** | **Upper** | **Value** | **Lower** | **Upper** | **Value** | **Lower** | **Upper** | **Value** | **Lower** | **Upper** |  |  |
| **GI** | **Appendicitis** | <5 | 519.5 | 173.9 | 823.3 | 489.4 | 303.9 | 806.3 | 75.6 | 52.8 | 113.3 | 1084.5 | 530.6 | 1742.9 |  |  |
|  |  | 5-9 | 320.1 | 165.7 | 480.3 | 508.3 | 362.5 | 662.4 | 100.0 | 81.5 | 126.2 | 928.4 | 609.7 | 1268.9 |  |  |
|  |  | 10-14 | 274.9 | 151.1 | 420.2 | 556.5 | 399.2 | 694.4 | 96.9 | 80.9 | 116.7 | 928.3 | 631.2 | 1231.3 |  |  |
|  |  | 15-19 | 211.1 | 122.2 | 330.4 | 1486.0 | 1126.9 | 1877.4 | 112.3 | 95.8 | 132.8 | 1809.4 | 1344.9 | 2340.6 |  |  |
|  |  | ***Sub-total*** | ***1325.6*** | ***612.9*** | ***2054.2*** | ***3040.2*** | ***2192.5*** | ***4040.5*** | ***384.8*** | ***311.0*** | ***489.0*** | ***4750.6*** | ***3116.4*** | ***6583.7*** | ***16.7*** | ***1.5*** |
|  | **Inguinal, femoral, and abdominal hernia** | <5 | 967.2 | 512.6 | 1410.6 | 1046.2 | 750.2 | 1406.2 | 118.7 | 84.8 | 180.6 | 2132.1 | 1347.6 | 2997.4 |  |  |
|  |  | 5-9 | 45.0 | 22.9 | 81.3 | 119.8 | 60.9 | 179.4 | 12.6 | 9.4 | 17.0 | 177.4 | 93.2 | 277.7 |  |  |
|  |  | 10-14 | 31.0 | 16.7 | 57.1 | 85.7 | 52.6 | 111.0 | 7.4 | 5.6 | 9.9 | 124.1 | 74.9 | 178.0 |  |  |
|  |  | 15-19 | 35.7 | 19.6 | 62.6 | 171.7 | 122.7 | 235.8 | 11.1 | 9.0 | 14.3 | 218.5 | 151.3 | 312.7 |  |  |
|  |  | ***Sub-total*** | ***1078.9*** | ***571.8*** | ***1611.6*** | ***1423.4*** | ***986.4*** | ***1932.4*** | ***149.8*** | ***108.8*** | ***221.8*** | ***2652.1*** | ***1667.0*** | ***3765.8*** | ***9.3*** | ***0.8*** |
|  | **Paralytic ileus and intestinal obstruction** | <5 | 4324.9 | 2983.5 | 6517.3 | 7844.5 | 4674.6 | 12534.1 | 1706.3 | 1296.9 | 2217.1 | 13875.7 | 8955.0 | 21268.5 |  |  |
|  |  | 5-9 | 614.2 | 381.8 | 910.0 | 1437.6 | 877.2 | 2080.1 | 137.4 | 116.4 | 157.8 | 2189.2 | 1375.4 | 3147.9 |  |  |
|  |  | 10-14 | 538.9 | 383.6 | 741.3 | 1001.8 | 666.4 | 1356.3 | 80.0 | 68.8 | 91.6 | 1620.7 | 1118.8 | 2189.2 |  |  |
|  |  | 15-19 | 674.5 | 497.1 | 901.2 | 2567.4 | 1723.4 | 3341.6 | 84.5 | 72.2 | 98.0 | 3326.4 | 2292.7 | 4340.8 |  |  |
|  |  | ***Sub-total*** | ***6152.5*** | ***4246.0*** | ***9069.8*** | ***12851.3*** | ***7941.6*** | ***19312.1*** | ***2008.2*** | ***1554.3*** | ***2564.5*** | ***21012.0*** | ***13741.9*** | ***30946.4*** | ***73.9*** | ***6.7*** |
|  | **GI Total** |  | **8557.0** | **5430.7** | **12735.6** | **17314.9** | **11120.5** | **25285.0** | **2542.8** | **1974.1** | **3275.3** | **28414.7** | **18525.3** | **41295.9** | ***100.0*** | ***9.0*** |
| Maternal- Fetal | Maternal abortion and miscarriage | <5 | -- | -- | -- | -- | -- | -- | -- | -- | -- | -- | -- | -- |  |  |
|  |  | 5-9 | -- | -- | -- | -- | -- | -- | -- | -- | -- | -- | -- | -- |  |  |
|  |  | 10-14 | 145.5 | 96.0 | 213.2 | 60.6 | 38.8 | 97.1 | 2.4 | 1.9 | 3.0 | 208.5 | 136.7 | 313.3 |  |  |
|  |  | 15-19 | 1186.6 | 915.2 | 1486.4 | 670.7 | 497.3 | 912.9 | 41.4 | 33.5 | 50.2 | 1898.7 | 1446.0 | 2449.5 |  |  |
|  |  | ***Sub-total*** | ***1332.1*** | ***1011.2*** | ***1699.6*** | ***731.3*** | ***536.1*** | ***1010.0*** | ***43.8*** | ***35.4*** | ***53.2*** | ***2107.2*** | ***1582.7*** | ***2762.8*** | ***1.0*** | ***0.7*** |
|  | Maternal  hemorrhage | <5 | -- | -- | -- | -- | -- | -- | -- | -- | -- | -- | -- | -- |  |  |

|  |  | 5-9 | -- | -- | -- | -- | -- | -- | -- | -- | -- | -- | -- | -- |  |  |
| --- | --- | --- | --- | --- | --- | --- | --- | --- | --- | --- | --- | --- | --- | --- | --- | --- |
|  |  | 10-14 | 17.4 | 11.7 | 25.4 | 44.4 | 29.3 | 64.9 | 1.7 | 1.3 | 2.1 | 63.5 | 42.3 | 92.4 |  |  |
|  |  | 15-19 | 321.6 | 252.4 | 394.4 | 1037.3 | 800.5 | 1328.0 | 34.6 | 29.4 | 41.1 | 1393.5 | 1082.3 | 1763.5 |  |  |
|  |  | ***Sub-total*** | ***339.0*** | ***264.1*** | ***419.8*** | ***1081.7*** | ***829.8*** | ***1392.9*** | ***36.3*** | ***30.7*** | ***43.2*** | ***1457.0*** | ***1124.6*** | ***1855.9*** | ***0.7*** | ***0.5*** |
|  | Maternal obstructed labor and uterine rupture | <5 | -- | -- | -- | -- | -- | -- | -- | -- | -- | -- | -- | -- |  |  |
|  |  | 5-9 | -- | -- | -- | -- | -- | -- | -- | -- | -- | -- | -- | -- |  |  |
|  |  | 10-14 | 13.8 | 8.5 | 23.0 | 17.3 | 11.1 | 26.7 | 1.2 | 0.8 | 1.9 | 32.3 | 20.4 | 51.6 |  |  |
|  |  | 15-19 | 268.2 | 196.9 | 358.9 | 519.8 | 366.5 | 714.5 | 27.2 | 21.6 | 34.9 | 815.2 | 585.0 | 1108.3 |  |  |
|  |  | ***Sub-total*** | ***282.0*** | ***205.4*** | ***381.9*** | ***537.1*** | ***377.6*** | ***741.2*** | ***28.4*** | ***22.4*** | ***36.8*** | ***847.5*** | ***605.4*** | ***1159.9*** | ***0.4*** | ***0.3*** |
|  | Neonatal encephalopat hy due to birth asphyxia and trauma | <5 | 62753.7 | 48925.6 | 78778.6 | 133587.5 | 111932.3 | 157461.0 | 2747.1 | 2353.3 | 3203.7 | 199088.3 | 163211.2 | 239443.3 |  |  |
|  |  | 5-9 | -- | -- | -- | -- | -- | -- | -- | -- | -- | -- | -- | -- |  |  |
|  |  | 10-14 | -- | -- | -- | -- | -- | -- | -- | -- | -- | -- | -- | -- |  |  |
|  |  | 15-19 | -- | -- | -- | -- | -- | -- | -- | -- | -- | -- | -- | -- |  |  |
|  |  | ***Sub-total*** | ***62753.7*** | ***48925.6*** | ***78778.6*** | ***133587.5*** | ***111932.3*** | ***157461.0*** | ***2747.1*** | ***2353.3*** | ***3203.7*** | ***199088.3*** | ***163211.2*** | ***239443.3*** | ***97.8*** | ***63.3*** |
|  | **Maternal- Neonatal**  **Total** |  | **64706.8** | **50406.3** | **81279.9** | **135937.6** | **113675.8** | **160605.1** | **2855.6** | **2441.8** | **3336.9** | **203500.0** | **166523.9** | **245221.9** | **100.0** | **64.7** |
| Injuries | Adverse effects of medical treatment Total | <5 | 3433.8 | 2231.1 | 4919.0 | 3635.6 | 2576.4 | 5032.9 | 372.2 | 277.4 | 500.5 | 7441.6 | 5084.9 | 10452.4 |  |  |
|  |  | 5-9 | 402.2 | 271.1 | 597.5 | 554.4 | 414.0 | 720.7 | 81.0 | 70.9 | 103.9 | 1037.6 | 756.0 | 1422.1 |  |  |
|  |  | 10-14 | 247.8 | 151.1 | 452.4 | 407.7 | 294.9 | 505.1 | 45.9 | 41.3 | 59.5 | 701.4 | 487.3 | 1017.0 |  |  |
|  |  | 15-19 | 251.4 | 136.0 | 525.6 | 448.9 | 308.7 | 554.6 | 49.4 | 41.0 | 58.8 | 749.7 | 485.7 | 1139.0 |  |  |
|  |  | ***Sub-total*** | ***4335.2*** | ***2789.3*** | ***6494.5*** | ***5046.6*** | ***3594.0*** | ***6813.3*** | ***548.5*** | ***430.6*** | ***722.7*** | ***9930.3*** | ***6813.9*** | ***14030.5*** | ***12.0*** | ***3.2*** |
|  | Exposure to mechanical forces | <5 | 1140.5 | 462.7 | 2010.8 | 1996.0 | 760.7 | 3271.8 | 746.1 | 397.5 | 995.3 | 3882.6 | 1620.9 | 6277.9 |  |  |
|  |  | 5-9 | 198.5 | 117.0 | 304.3 | 480.3 | 289.9 | 672.3 | 326.0 | 231.7 | 388.9 | 1004.8 | 638.6 | 1365.5 |  |  |
|  |  | 10-14 | 127.1 | 78.1 | 196.6 | 290.7 | 165.1 | 406.4 | 222.8 | 158.5 | 266.7 | 640.6 | 401.7 | 869.7 |  |  |
|  |  | 15-19 | 222.3 | 149.4 | 349.6 | 546.2 | 317.6 | 726.0 | 587.0 | 409.6 | 700.2 | 1355.5 | 876.6 | 1775.8 |  |  |
|  |  | ***Sub-total*** | ***1688.4*** | ***807.2*** | ***2861.3*** | ***3313.2*** | ***1533.3*** | ***5076.5*** | ***1881.9*** | ***1197.3*** | ***2351.1*** | ***6883.5*** | ***3537.8*** | ***10288.9*** | ***8.3*** | ***2.2*** |
|  | Falls | <5 | 781.1 | 469.9 | 1208.6 | 3301.4 | 1991.6 | 4674.4 | 1616.9 | 1096.4 | 1978.9 | 5699.4 | 3557.9 | 7861.9 |  |  |

|  |  | 5-9 | 425.2 | 272.3 | 627.1 | 1181.0 | 839.9 | 1571.4 | 763.3 | 562.9 | 901.2 | 2369.5 | 1675.1 | 3099.7 |  |  |
| --- | --- | --- | --- | --- | --- | --- | --- | --- | --- | --- | --- | --- | --- | --- | --- | --- |
|  |  | 10-14 | 319.9 | 222.3 | 444.4 | 1082.7 | 775.3 | 1392.4 | 522.2 | 376.1 | 600.0 | 1924.8 | 1373.7 | 2436.8 |  |  |
|  |  | 15-19 | 17.0 | -22.1 | 47.6 | 844.3 | 636.1 | 1096.1 | 679.4 | 482.9 | 785.1 | 1540.7 | 1096.9 | 1928.8 |  |  |
|  |  | ***Sub-total*** | ***1543.2*** | ***942.4*** | ***2327.7*** | ***6409.4*** | ***4242.9*** | ***8734.3*** | ***3581.8*** | ***2518.3*** | ***4265.2*** | ***11534.4*** | ***7703.6*** | ***15327.2*** | ***13.9*** | ***3.7*** |
|  | Fire, heat, and hot substances | <5 | 2656.7 | 1710.1 | 4040.0 | 3606.8 | 2131.2 | 5426.5 | 435.1 | 260.3 | 662.8 | 6698.6 | 4101.6 | 10129.3 |  |  |
|  |  | 5-9 | 374.3 | 227.0 | 568.4 | 674.8 | 287.9 | 1061.0 | 143.3 | 86.3 | 211.5 | 1192.4 | 601.2 | 1840.9 |  |  |
|  |  | 10-14 | 172.3 | 113.0 | 253.5 | 662.6 | 326.0 | 1028.3 | 74.6 | 45.6 | 108.8 | 909.5 | 484.6 | 1390.6 |  |  |
|  |  | 15-19 | 162.3 | 106.4 | 235.8 | 1554.3 | 796.9 | 2396.5 | 177.7 | 123.9 | 227.5 | 1894.3 | 1027.2 | 2859.8 |  |  |
|  |  | ***Sub-total*** | ***3365.6*** | ***2156.5*** | ***5097.7*** | ***6498.5*** | ***3542.0*** | ***9912.3*** | ***830.7*** | ***516.1*** | ***1210.6*** | ***10694.8*** | ***6214.6*** | ***16220.6*** | ***12.9*** | ***3.4*** |
|  | Interpersonal violence | <5 | 551.8 | 261.0 | 872.7 | 138.5 | -184.0 | 485.2 | -55.1 | -101.8 | -0.5 | 635.2 | -24.7 | 1357.4 |  |  |
|  |  | 5-9 | 174.6 | 83.2 | 264.4 | 341.5 | 160.9 | 530.5 | 265.9 | 232.8 | 301.5 | 782.0 | 476.8 | 1096.4 |  |  |
|  |  | 10-14 | 212.2 | 123.1 | 297.3 | 449.1 | 274.6 | 646.6 | 524.0 | 462.9 | 594.9 | 1185.4 | 860.5 | 1538.8 |  |  |
|  |  | 15-19 | 812.0 | 527.2 | 1131.5 | 468.2 | 32.3 | 939.0 | 4110.6 | 3680.0 | 4575.3 | 5390.8 | 4239.5 | 6645.8 |  |  |
|  |  | ***Sub-total*** | ***1750.5*** | ***994.5*** | ***2565.9*** | ***1397.4*** | ***283.8*** | ***2601.4*** | ***4845.5*** | ***4273.9*** | ***5471.1*** | ***7993.4*** | ***5552.1*** | ***10638.4*** | ***9.7*** | ***2.5*** |
|  | Non- venomous animal contact | <5 | 437.5 | 297.5 | 650.2 | 299.2 | 208.4 | 423.6 | 27.3 | 19.5 | 37.3 | 763.9 | 525.4 | 1111.2 |  |  |
|  |  | 5-9 | 142.4 | 92.6 | 218.7 | 103.3 | 76.7 | 141.2 | 30.2 | 25.1 | 36.8 | 275.8 | 194.4 | 396.7 |  |  |
|  |  | 10-14 | 84.9 | 58.5 | 133.1 | 73.7 | 52.1 | 106.0 | 13.7 | 11.2 | 17.5 | 172.3 | 121.8 | 256.6 |  |  |
|  |  | 15-19 | 59.1 | 41.9 | 89.2 | 52.0 | 38.6 | 72.3 | 15.0 | 12.2 | 18.2 | 126.2 | 92.8 | 179.7 |  |  |
|  |  | ***Sub-total*** | ***723.8*** | ***490.4*** | ***1091.2*** | ***528.1*** | ***375.8*** | ***743.0*** | ***86.2*** | ***68.1*** | ***109.9*** | ***1338.2*** | ***934.3*** | ***1944.1*** | ***1.6*** | ***0.4*** |
|  | Road injuries | <5 | 4103.6 | 2739.3 | 6531.0 | 6121.0 | 4586.5 | 8338.2 | 2484.6 | 1981.0 | 3001.6 | 12709.3 | 9306.8 | 17870.8 |  |  |
|  |  | 5-9 | 2314.3 | 1664.2 | 3279.2 | 4722.2 | 3575.3 | 6111.1 | 2542.7 | 2246.8 | 2872.2 | 9579.2 | 7486.3 | 12262.5 |  |  |
|  |  | 10-14 | 1503.6 | 1078.4 | 2044.6 | 3596.1 | 2662.4 | 4591.3 | 1730.0 | 1527.5 | 1950.5 | 6829.7 | 5268.3 | 8586.4 |  |  |
|  |  | 15-19 | 1356.0 | 628.2 | 2196.0 | 280.8 | -1128.1 | 1954.4 | 1926.7 | 1428.2 | 2617.4 | 3563.4 | 928.2 | 6767.8 |  |  |
|  |  | ***Sub-total*** | ***9277.5*** | ***6110.1*** | ***14050.8*** | ***14720.1*** | ***9696.0*** | ***20995.1*** | ***8684.0*** | ***7183.4*** | ***10441.7*** | ***32681.6*** | ***22989.6*** | ***45487.6*** | ***39.5*** | ***10.4*** |
|  | Other transport injuries | <5 | 207.5 | 69.7 | 328.9 | 327.7 | 207.2 | 447.5 | 36.7 | 27.9 | 46.3 | 571.9 | 304.8 | 822.7 |  |  |
|  |  | 5-9 | 82.4 | 42.9 | 122.9 | 138.4 | 82.6 | 199.2 | 19.1 | 13.8 | 25.2 | 240.0 | 139.2 | 347.2 |  |  |

|  |  | 10-14 | 45.9 | 22.1 | 76.2 | 184.0 | 88.7 | 271.3 | -24.4 | -27.6 | -21.2 | 205.4 | 83.1 | 326.2 |  |  |
| --- | --- | --- | --- | --- | --- | --- | --- | --- | --- | --- | --- | --- | --- | --- | --- | --- |
|  |  | 15-19 | 69.2 | 25.8 | 172.4 | 554.7 | 289.1 | 767.1 | -3.3 | -18.7 | 14.4 | 620.6 | 296.3 | 953.9 |  |  |
|  |  | ***Sub-total*** | ***405.1*** | ***160.5*** | ***700.3*** | ***1204.7*** | ***667.6*** | ***1685.1*** | ***28.1*** | ***-4.6*** | ***64.7*** | ***1637.9*** | ***823.4*** | ***2450.1*** | ***2.0*** | ***0.5*** |
|  | **Injury Total** |  | **23089.3** | **14450.9** | **35189.4** | **39118.1** | **23935.4** | **56561.0** | **20486.8** | **16183.1** | **24637.0** | **82694.1** | **54569.4** | **116387.4** | **100.0** | **26.3** |
| **Age group totals** | | <5 | 81877.7 | 60837.0 | 108091.0 | 162393.8 | 129939.0 | 200307.8 | 10311.5 | 7746.1 | 12936.9 | 254583.1 | 198522.0 | 321335.7 | 80.9 |  |
|  |  | 5-9 | 5093.2 | 3340.6 | 7454.1 | 10261.6 | 7027.7 | 13929.3 | 4421.5 | 3677.6 | 5142.2 | 19776.3 | 14045.9 | 26525.6 | 6.3 |  |
|  |  | 10-14 | 3735.2 | 2514.1 | 5378.3 | 8512.8 | 5836.5 | 11297.8 | 3298.5 | 2754.7 | 3801.9 | 15546.5 | 11105.3 | 20478.0 | 4.9 |  |
|  |  | 15-19 | 5647.0 | 3596.2 | 8281.5 | 11202.3 | 5928.5 | 16916.2 | 7853.7 | 6420.6 | 9368.2 | 24703.0 | 15945.4 | 34565.9 | 7.9 |  |
| **TOTAL NUMBER OF AVERTABLE DEATHS** | | | **96353.1** | **70287.9** | **129204.9** | **192370.6** | **148731.7** | **242451.1** | **25885.2** | **20599.0** | **31249.2** | **314608.8** | **239618.6** | **402905.2** | **100.0** | **100.0** |
| **% of total by World Bank groupings** | | | **30.6** |  |  | **61.1** |  |  | **8.2** |  |  | **100.0** |  |  |  |  |
