# Supplementary material for: Estimates of Treatable Deaths Within the First 20 Years of Life from Scaling Up Surgical Care at First-Level Hospitals in Low- and Middle-Income Countries
Source: World J Surg. 2022 Jun 30;46(9):2114–22. doi: 10.1007/s00268-022-06622-w (PMC9334432; doi:10.1007/s00268-022-06622-w)
Supplement: Supplementary file 4 — Supplementary file4 (DOCX 8 KB) [file 268_2022_6622_MOESM4_ESM.docx]

Additional file 4_ Summary of maternal and neonatal deaths in the 2019 GBD Study

|  |  |  |  |  |  |  |
| --- | --- | --- | --- | --- | --- | --- |
| Sum of val2 | Column Labels |  |  |  |  | **LMICs sum** |
| **Row Labels** | **World Bank High Income** | **World Bank Low Income** | **World Bank Lower Middle Income** | **World Bank Upper Middle**  **Income** | **Grand Total** |  |
| **Maternal and neonatal disorders** | 24322.8 | 485772.4 | 1217058.3 | 174148.8 | 1901302.3 | 1,876,979.4 |
| **10 to 14** | 4.1 | 449.7 | 473.9 | 49.6 | 977.3 | 973.2 |
| **15 to 19** | 69.3 | 6603.0 | 11314.1 | 974.5 | 18960.8 | 18,891.6 |
| **Under 5** | 24249.4 | 478719.7 | 1205270.3 | 173124.7 | 1881364.1 | 1,857,114.7 |
| **Maternal disorders** | 73.4 | 7052.7 | 11788.0 | 1024.1 | 19938.2 | 19,864.8 |
| **10 to 14** | 4.1 | 449.7 | 473.9 | 49.6 | 977.3 | 973.2 |
| **15 to 19** | 69.3 | 6603.0 | 11314.1 | 974.5 | 18960.8 | 18,891.6 |
| **Neonatal disorders** | 24249.4 | 478719.7 | 1205270.3 | 173124.7 | 1881364.1 | 1,857,114.7 |
| **Under 5** | 24249.4 | 478719.7 | 1205270.3 | 173124.7 | 1881364.1 | 1,857,114.7 |
| **Neonatal encephalopathy due to birth asphyxia and trauma** | 4142.6 | 168306.3 | 355113.1 | 39136.8 | 566698.7 | 562,556.2 |
| **Under 5** | 4142.6 | 168306.3 | 355113.1 | 39136.8 | 566698.7 | 562,556.2 |
| **Grand Total** | 52788.2 | 1139851.1 | 2789229.6 | 387434.3 | 4369303.3 | 4,316,515.0 |
